# Sebaceous carcinoma of the lip: a case report and review of the literature

**DOI:** 10.1186/s13256-022-03435-2

**Published:** 2022-06-18

**Authors:** Michele Di Cosola, Francesca Spirito, Mariateresa Ambrosino, Pasquale Somma, Andrea Santarelli, Stefania Staibano, Lorenzo Lo Muzio

**Affiliations:** 1 Department of Clinical and Experimental Medicine, University of Foggia, Via Carelli 28, 71100 Foggia, Italy; 2 Department of Biomorphological and Functional Sciences, University of Naples “Federico II”, 80121 Naples, Italy; 3 Dipartimento di Scienze Cliniche Specialistiche e Odontostomatologiche, Università Politecnica delle Marche, 60121 Ancona, Italy

**Keywords:** Sebaceous carcinoma, Lower lip, Immunohistochemistry, Case report

## Abstract

**Background:**

Sebaceous carcinoma is a very rare, aggressive, malignant tumor arising in the adnexal epithelium of the sebaceous gland. Sebaceous carcinoma in the oral cavity is extremely rare, with only 14 cases reported in literature. We reported the fourth case of sebaceous carcinoma involving the lip

**Case presentation:**

A 71-year-old Caucasian male smoker presented an ulcerated lesion in the lateral region of the lower lip. The patient stated that the lesion had been present for 1 year. The past medical history was unremarkable. Extraoral examination revealed a markedly ulcerated, exophytic, irregularly shaped, indurated mass of the lower right labial region, measuring 1.8 cm in size. The nodular lesion, located at the point of transition between mucosa and skin, showed a central ulceration. No other intraoral lesions were identified. The clinical differential diagnosis included squamous cell carcinoma, basal cell carcinoma with sebaceous differentiation, and salivary gland neoplasms. Operation was performed under local anesthesia. On histopathological examination, the tumor was composed by nodules or sheet of cells separated by a fibrovascular stroma. The neoplastic tissue was deeply infiltrating, involving the submucosa and even the underlying muscle. Neoplastic cells showed a range of sebaceous differentiation with finely vacuolated rather than clear cytoplasm. Neoplastic cells were positive for S-100 protein and epithelial membrane antigen, but negative for carcinoembryonic antigen. Based on these findings, a diagnosis of sebaceous carcinoma of the lower lip was rendered.

**Conclusion:**

The histogenesis, differential diagnosis, and clinicopathological conditions of this disease according to literature are reviewed. Sebaceous carcinoma should be distinguished from other tumors full of vacuolated clear cells. A periodic acid-Schiff stain and immunohistochemical stain for Ki-67, P53, cytokeratin, S-100, epithelial membrane antigen, and androgen receptor can be useful for the diagnosis.

## Introduction

Sebaceous carcinoma (SC) is a rare neoplasm. To date, fewer than 400 cases have been reported in literature. Due to its low incidence and not universally accepted histopathological classification, it can present diagnostic problems [1]. Generally, the lesions arise in the meibomian glands of the eyelid. However, extraocular localization in the head and neck region has also been reported [1–3]. The salivary glands too are considered an uncommon site, even if some cases arising in the parotid gland have been recognized [4]. While several reports document sebaceous adenomas arising from sebaceous glands of the oral cavity, oral sebaceous carcinomas are extremely rare.

Sebaceous glands in the oral mucosa are widely found in approximately 80% of adults and are called Fordyce granules [5, 6]. Due to their high incidence rate, Fordyce granules are considered a normal anatomic variation [5, 6]. These granules appear as small asymptomatic yellow–white papules or granules in the oral mucosa. It seems that these intraoral sebaceous glands can rarely give rise to a variety of sebaceous neoplasms, such as sebaceous carcinoma [7]. Since 1991, when Damm *et al.* described the first known report in the English-language literature of sebaceous carcinoma presenting as an intraoral tumor [8], only 14 cases have been described (Table 1) [2, 3, 7–18].

**Table 1:** Reported cases of primary intraoral sebaceous carcinoma

	Authors	Year	Country	Sex	Age (years)	Smoker	Site	Size (cm)	Case	Tumor involving other structures	Treatment	Follow-up (years)
1	Damm *et al.* [8]	1991	USA	M	53	Unknown	Buccal mucosa (parotid duct)	3	Intraoral sebaceous carcinoma	No	Excision	5
2	Abuzeid *et al.* [9]	1996	Saudi Arabia	F	11	No	Buccal mucosa	3	Intraoral sebaceous carcinoma	Submandibular salivary gland, two lymph nodes	Excision	2
3	Liu *et al.* [10]	1997	Taiwan	M	68	Unknown	Buccal mucosa	2.5	Sebaceous carcinoma of buccal mucosa	No	Excision	3
4	Li *et al.* [11]	1997	Japan	M	78	Yes	Buccal mucosa	3.5	Oral sebaceous carcinoma	Surrounding muscle	Excision	6
5	Handschel *et al.* [3]	2003	Germany	F	80	No	Anterior floor of the mouth	1.5	Intraoral sebaceous carcinoma	No	Excision	1
6	Alawi *et al.* [2]	2005	USA	M	66	Yes	Upper lip	1.5	Sebaceous carcinoma of the oral mucosa	No	Excision	1
7	Innocenzi *et al.* [12]	2005	Italy	F	68	No	Upper lip	2	Sebaceous carcinoma	No	Excision	3
8	Gomes *et al.* [13]	2007	Brazil	M	55	Yes	Floor of mouth	Not reported	Intraoral sebaceous carcinoma	Mandible body and ramus, masseter muscle	Excision, chemotherapy, radiotherapy	1
9	Wang *et al.* [14]	2010	USA	M	50	No	Buccal mucosa	4.6	Sebaceous carcinoma of the oral cavity	No	Excision + radiation therapy	<1
10	Oshiro *et al.* [15]	2010	Japan	M	66	Unknown	Tongue and dorsum	2.5	Primary sebaceous carcinoma of the tongue	Cervical lymph nodes bilaterally, lung	Intraarterial chemotherapy/radiation	Died after 17 months
11	Rowe *et al.* [16]	2016	USA	M	76	Yes	Anterior maxillary gingiva	3	Intraoral sebaceous carcinoma metastatic to the lung and subcutis	Skin of thigh/buttocks/lungs	Excision + chemotherapy	<1
12	Greenall *et al.* [17]	2015	UK	M	81	Unknown	Right upper lip	Not reported	sebaceous carcinoma	Soft tissues of the right upper lip, buccal space, retromolar trigone (Fig. 2), pterygopalatine fossa, and apex of the infratemporal fossa	Palliative radiotherapy	Not reported
13	Wetzel *et al.* [18]	2015	USA	M	75	Yes	Maxillary gingiva	Not reported	Sebaceous carcinoma	No	Excision	Not reported
14	Lu *et al.* [7]	2021	China	F	62	Unknown	Soft palate	2	Intraoral sebaceous carcinoma	No	Excision	Not reported
15	Present case	2021	Italy	M	71	Yes	Lip	1.8	Sebaceous carcinoma	No	Excision	3

Regardless of the localization, sebaceous malignancies must be considered aggressive neoplasms with potential for regional and distant metastases. Treatment of this tumor points to a wide surgical excision with safe margins. Eventually, implicated regional lymph nodes have to be excised too [19, 20]. Disagreement still exists concerning the efficacy of postsurgical irradiation and/or chemotherapy [1, 19, 20].

We reported herein a case of SC arising in the lateral edge of the lower lip in a 71-year-old man. To the best of our knowledge, this is the fourth case described in lips.

## Case report

**Fig. 1:**
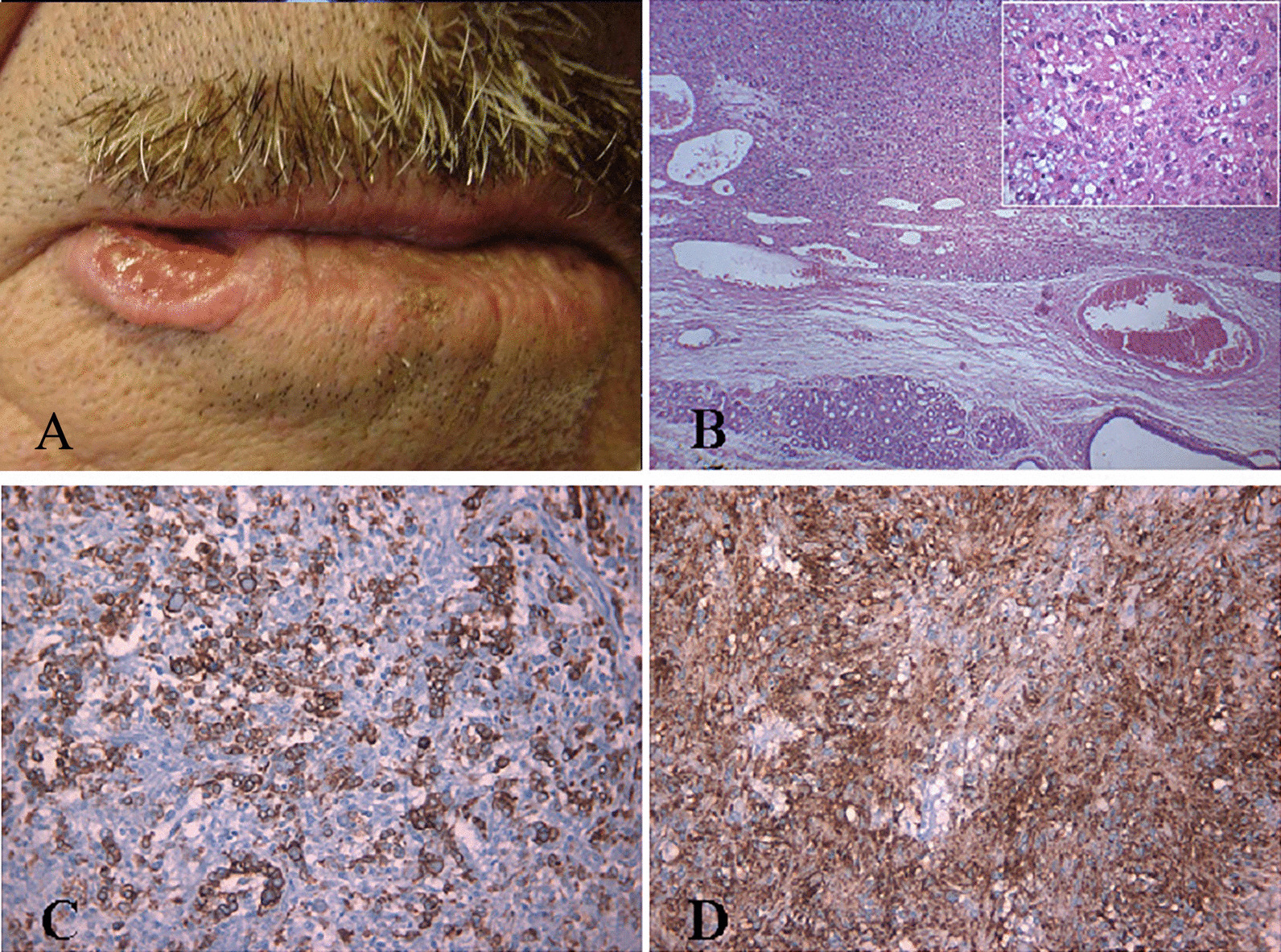
**A** Clinical features of the lesion before surgical treatment; **B** Submucosal sebaceous carcinoma showing an infiltrative pattern (E&E ×40) and nests of atypical clear cells with scattering squamous differentiation (inset, E&E ×400); **C** The neoplastic cells were positive for S-100 (Avidin/Biotin Method or ABC ×200); **D** The neoplastic cells were positive for epithelial membrane antigen (ABC ×200)

A 71-year-old Caucasian male smoker presented an ulcerated lesion in the lateral region of the lower lip (Fig. 1A). He had past medical history of tobacco abuse with 15 cigarettes/day smoking history without any other remarkable medical or family history. The patient stated that the lesion had been present for 1 year, but he did not complain of any pain symptoms. No previous trauma in face or mouth was recalled. Extraoral examination revealed a markedly ulcerated, exophytic, irregularly shaped, indurated mass of the lower right labial region, measuring 1.8 cm in size. The nodular lesion, located at the point of transition between mucosa and skin, showed a central ulceration. No other intraoral lesions were identified.

On palpation, the neck was soft with full range of motion, and there was no evidence of lymphadenopathy or tenderness. The patient was in good health and had no evidence of disease.

The clinical differential diagnosis included squamous cell carcinoma, basal cell carcinoma with sebaceous differentiation (BCCSD), and salivary gland neoplasms.

Routine laboratory analysis was performed before surgery, and no abnormal findings were detected.

The operation was performed under local anesthesia. The lesion was removed with 0.5 cm of free margins and a W-shaped wedge. The defect was primarily closed. The postoperative course was uneventful.

Four-micron-thick serial sections were obtained from formalin-fixed, paraffin-embedded surgical specimen. One section was stained with hematoxylin–eosin. Immunohistochemistry was performed on the remaining serial sections using the labeled streptavidin-biotin complex system (DAKO) to study the expression of epithelial membrane antigen (EMA), S-100 protein, and carcinoembryonic antigen (CEA), using the following primary antibodies: anti-EMA (057 M Biogenex; 1:100), anti-S100 (058 P Biogenex; 1:100), and anti-CEA (365 M Biogenex; 1:100).

On histopathological examination, the tumor was composed by nodules or sheet of cells separated by a fibrovascolar stroma. The neoplastic tissue was deeply infiltrating, involving the submucosa and even the underlying muscle (Fig. 1B). Neoplastic cells showed a range of sebaceous differentiation with finely vacuolated rather than clear cytoplasm. Also, areas with squamous differentiation were present. Large nuclei with large nucleoli, as well as scattered and atypical mitoses, were observed (Fig. 1B, inset). Neoplastic cells were positive for S-100 protein (Fig. 1C) and EMA (Fig. 1D), but negative for CEA. Based on these findings, a diagnosis of sebaceous carcinoma of the lower lip was rendered. Following the diagnosis, the patient underwent a complete clinical and radiographic evaluation to identify any regional or distant metastases. Because no metastases were detected, no further treatment was deemed necessary. Follow-up visits were performed every 6 months for 3 years.

All clinical and diagnostic steps are summarized in Table 2.

**Table 2:** Clinical and diagnostic steps

1. Appearance of an ulcerated lesion in the lateral region of the lower lip;
2. After the lesion had been present for 1 year, the patient presented for specialist medical consultation;
3. The patient underwent full examination of oral and extraoral tissues and a markedly ulcerated, exophytic, irregularly shaped, indurated mass of the lower right labial region, measuring 1.8 cm in size, was revealed;
4. An excisional biopsy with 0.5 cm of free margins and W-shaped wedge was performed;
5. Four-micron-thick serial sections were obtained from a formalin-fixed, paraffin-embedded surgical specimen;
6. One section was stained with hematoxylin–eosin for histopathological examination;
7. Immunohistochemistry was performed to study the expression of EMA and CEA;
8. Based on the findings, a diagnosis of sebaceous carcinoma of the lower lip was rendered;
9. Following the diagnosis, the patient underwent a complete clinical and radiographic evaluation to identify any regional or distant metastases.

## Discussion

Glands with sebaceous differentiation are often found in the oral cavity, and sebaceous differentiation may also be detected in the major salivary glands. Nevertheless, SC is rare in these anatomic sites [3, 21]. Sebaceous glands may be present in the lip, as described by Miles [21]. Literature data indicate that extraocular sebaceous carcinomas are less aggressive than orbital ones. Furthermore, the former rarely metastasized [19]. However a recent study, involving 2422 cases of SC over 10 years, showed that, among extraocular head and neck SC cases, none of the orbital tumor metastasized to the locoregional lymph nodes whereas two of five cases of extraocular SC metastasized to locoregional lymph nodes [20].

A comprehensive literature review identified only 15 cases of intraoral SC, of which the primary sites reported were the buccal mucosa, mouth floor, upper labial mucosa, palate, gingiva, and tongue. The majority of cases of SC occur on the buccal mucosa (5/15, 22.22%). Other sites include the labial mucosa with 4/15 (26%), anterior floor of mouth with 2/15 (13%), gingiva with 2/15 (13%), palate with 1/15 (7%), and tongue with 1/15 (7%), with 11/15 being men (73.3%) and 4/15 women (26.7%). The age ranged from 11 to 81 years old (mean 64 years). The reported size of 12 lesions ranged from 1.5 to 4.6 cm (mean 2.55 cm), while 6/15 cases (40%) showed involvement of contiguous sites or presence of metastasis to lymph nodes or lung. Data about smoking seem to be very interesting, with 6/10 being smokers (60%).

In the present case, a diagnosis of oral SC was made based on clinical detection of an ulcerated, exophytic, irregularly shaped, indurated mass on the lower right labial region and histopathological findings of nodules or sheet of cells separated by a fibrovascular stroma, deeply infiltrating, and neoplastic cells showing sebaceous and squamous differentiation. To the best of our knowledge, this is the fourth case of SC of the lip described in literature.

Although SC may be found among the multiple sebaceous neoplasms occurring in association with multiple visceral carcinomas in Muir–Torre syndrome [22], the lip was the only localization of SC in the present case.

SC must be distinguished from basal cell carcinoma with sebaceous differentiation (BCCSD). Basal cell carcinoma is characterized by superficial plate-like proliferation of basaloid and/or squamoid cells, with broad attachments to the overlying epidermis. The cells did not show high cytological atypia or atypical mitoses. Clusters of mature cells are abruptly interposed among otherwise typical basaloid cell nests, without transitional form [23, 24]. However, differential diagnosis could be particularly difficult because areas of sebaceous differentiation may have similar distribution in both lesions [25]. As in the present case, the involvement of epidermis or dermis favors the diagnosis of SC, but unfortunately is not invariably present [25].

The diagnosis may be facilitated by lipophylic stains on frozen sections or immune stains for EMA and S-100. While SC results diffusely positive for both previously mentioned antibodies, BCCSD shows reactivity only in areas with evident sebaceous differentiation [26–29]. SC must also be differentiated from squamous cell carcinoma (SCC) with hydropic degeneration. However, the latter shows a different pattern of growth. Moreover, cytological atypia and atypical mitoses are more evident and neoplastic cells are positive for cytokeratin and EMA, but negative for S-100 [26, 29].

Like other extremely rare neoplasms, the optimal treatment of SC is not fully conclusive. The therapeutic options range from wide excision to pre- and postoperative radiotherapy with or without chemotherapy [26]. In our case, wide excision of the lesion was performed without other postoperative therapy. The patient is alive without evidence of recurrence or metastases after 36 months of follow-up.

## Conclusion

A rare case of SC in a patient’s lip was observed. Radical surgery was carried out. The histogenesis, differential diagnosis, and clinicopathological conditions of this disease according to literature are reviewed. SC should be distinguished from other tumors full of vacuolated clear cells. Useful biomarkers to help verify the diagnosis can be Ki-67, P53, CK, PAS, S-100, EMA, and AR. Postoperative chemotherapy and radiotherapy were not adopted in the present case, as the postoperative pathology showed negative tumor margins and there was no evidence of lymph node metastasis; the patient was relatively satisfied with the surgery.

## Data Availability

Not applicable.
